# A New Method on Construction of Brain Effective Connectivity Based on Functional Magnetic Resonance Imaging

**DOI:** 10.1155/2022/4542106

**Published:** 2022-04-04

**Authors:** Jincan Zhang, Wenya Yang, Jiaofen Nan

**Affiliations:** ^1^School of Management Engineering, Zhengzhou University, Zhengzhou 450000, China; ^2^School of Computer and Communication Engineering, Zhengzhou University of Light Industry, Zhengzhou 450000, China

## Abstract

Most of the existing methods about the causal relationship based on functional magnetic resonance imaging (fMRI) data are either the hypothesis-driven methods or based on a linear model, which can result in the deviation for detecting the original brain activity. Therefore, it is necessary to develop a new method for detecting the effective connectivity (EC) of the brain activity by the nonlinear calculation. In this study, we firstly proposed a new technology evaluating effective connectivity of the human brain based on back-propagation neural network with nonlinear model, named EC-BP. Next, we simulated four time series for assessing the feasibility and accuracy of EC-BP compared to Granger causality analysis (GCA). Finally, the proposed EC-BP was applied to the brain fMRI from 60 healthy subjects. The results from the four simulated time series showed that the proposed EC-BP can detect the originally causal relationship, consistent with the actual causality. However, the GCA can not find nonlinear causality. Based on the analysis of the fMRI data from the healthy participants, EC-BP and GCA showed the huge differences in the top 50 connections in descending order of EC. EC-BP showed all ECs related to hippocampus and parahippocampus, whereas GCA showed most ECs related to the paracentral lobule, caudate, putamen, and pallidum, which represents the brain regions with most frequent information passing measured by different methods. The proposed EC-BP method can provide supplementary information to GCA, which will promote more comprehensive detection and evaluation of brain EC.

## 1. Introduction

The human brain has been considered as a complex neural network, performing multiple tasks and controlling human behavior [1–3]. Each task or behavior usually involved more than one region or even the whole brain [4]. Clarifying the behavior mechanism cannot be done without the study on functional segregation and integration of the brain. Functional segregation and integration [5] can be described through the brain connectivity, including functional connectivity (FC) and effective connectivity (EC). FC, widely being used to reveal the human brain function in health and disease [6–8], describes the temporal correlation between spatially remote brain areas [9]. However, although reflecting the intensity of interaction between different brain regions, FC could not exhibit the direction of information communication between different regions. Different from FC, EC described the causal influence that a brain site exerts over one other [9]. Therefore, EC can provide supplementary information to FC for the interaction between brain signals.

Several methods are commonly used to calculate EC based on functional magnetic resonance imaging (fMRI) data, including structural equation model (SEM) [10], multivariate autoregressive modeling (MAR) [11], and dynamic causal modeling (DCM) [12] and Granger causality analysis (GCA) [13]. However, SEM, MAR, and DCM belong to hypothesis-driven methods. In this way, the disadvantage of the model may lead to the wrong conclusions. Compared with the above methods, the GCA method might evaluate EC only by considering the time series of fMRI, which could overcome the limitation about the prior knowledge. Currently, GCA has been used for the studies on mild cognitive impairment [14], Alzheimer's disease [15], depression [16], insomnia [17], schizophrenia [18], and so on. However, GCA is based on a linear model and assume the fixed time lags during the information transmission [19, 20], diverged so greatly from the constant changes of propagation delay between brain signals [21, 22]. Therefore, it is necessary to develop a new method for detecting the EC of the nonlinear variables for the brain activity.

Brain EC is simply the causality between different regions of interest (ROIs) from the brain, representing the ability of one ROI predicting the other one. With the development of the neural network, deep learning, and machine learning, it is possible to evaluate EC of the brain by some nonlinear methods. Back-propagation neural network (BPNN), as a widely used neural network model, has been used for the prediction in many fields such as control science and engineering [23], chemical [24], and biomedical engineering [25], showing its good ability of prediction. Moreover, BPNN not only is a powerful data-driven computational tool but also can use a series of nonlinear functions to construct its model. Therefore, these aroused our speculation that BPNN with nonlinear model may assess EC with better performance.

In this study, we proposed a new technology evaluating EC of the human brain based on fMRI, namely, EC analysis based on BPNN with nonlinear model (EC-BP). First, the steps about EC-BP were described in detail. Second, we simulated the related data for assessing the feasibility and accuracy of EC-BP compared to GCA. Finally, EC-BP was applied to the brain EC analysis of the healthy human brain.

## 2. Material and Methods

### 2.1. Granger Causal Analysis

GCA, proposed by [26], is a commonly used method to study the causal relationship in complex systems. Generally, greater than 0.1 for GCA indicates that there is causal relationship. Here, GCA based on the *DynamicBC* toolbox [27] is used to evaluate the simulated data and fMRI of the healthy subjects, which will be compared with the proposed method.

### 2.2. Proposed EC-BP

**Figure 1:**
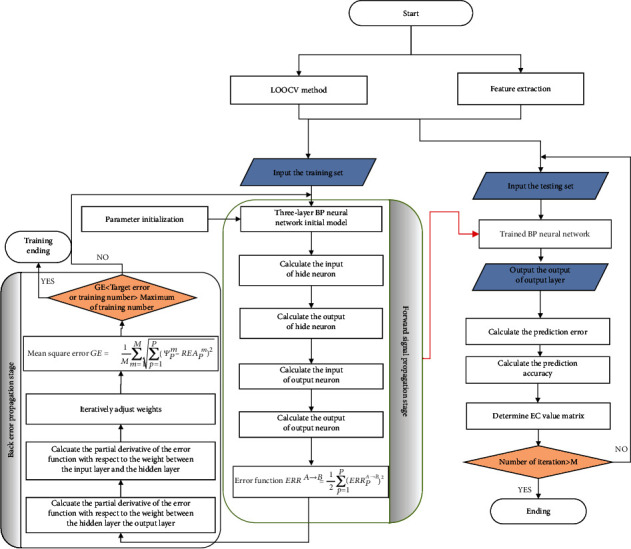
Flow chart of the method evaluating brain effective connectivity based on back-propagation neural network.

Based on the preprocessed resting-state fMRI data, the complete flow chart of the EC-BP method is shown in Figure 1. The main steps include feature extraction, construction on initial model of BPNN, BPNN training, and EC calculation via BPNN prediction.

#### 2.2.1. Feature Extraction

In order to predict the causal relationship between brain regions more accurately, we need to select one or more features of each brain region. The regional homogeneity (ReHo) proposed by Zang et al. in [28], as a typical feature of brain functional activity, has been widely used in fMRI, such as [29–31]. It is based on the assumption that the time series of adjacent voxels is similar, evaluating brain functional activity by calculating the Kendall's coefficient concordance (KCC) of voxels. Because of no special demands regarding samples, ReHo was calculated for our preliminary attempt, as follows.

The ReHo value can be calculated for each brain region, as shown as follows:

(1) $$\begin{array}{c} W_{b}=\frac{\sum \left({\text{Mask}}_{b}\cdot {\text{Matrix}}_{W}\right)}{V}, \end{array}$$

where *W*_*b*_ is the ReHo value of the *b*-th brain region, Mask_*b*_ is the mask matrix of the *b*-th brain area (the element value in the matrix is either 1 or 0, and 1 indicates that the corresponding brain voxel belongs to the brain region), *Σ* is the sum of all the elements from the matrix, and *V* is the number of voxels within the *b*-th brain region. Matrix_*w*_ is the ReHo matrix of all voxels in the whole brain, with each value corresponding ReHo of a voxel, calculated as follows:

(2) ReHov=12∑∑k=1KSτk2−n1/T∑τ=1T∑k=1KSτk2K2T3−T,

where ReHo_*v*_ represents the ReHo value of the*v*‐thvoxel (ranged from 0 to 1), *K* represents the number of neighbors of the *v*‐thvoxel in the brain plus 1 (*K* generally takes 7, 9, or 27, here *K* = 27), *Sτk* (*τ* =1,2,…*T*, *k* = 1, 2, ⋯*K*, and *T* is the number of time points of fMRI data) represents the sorting number of the *τ*‐th time point from *k*-th voxel, in ascending order of all values based on all time points for each voxel. The ReHo values were arranged in order of the number of brain regions for all subjects, constructing a *N* × *M* matrix, where *N* is the number of brain regions and *M* is the number of subjects.

#### 2.2.2. Constructing Initial Model of BPNN

The current study sets the number of layers of the network to 3 layers, including the input layer, the hidden layer, and the output layer. The number of neurons in the input layer is determined by the number of the selected features, while the number of neurons in the output layer is determined by the number of the predicting results. Therefore, the number of input neurons and output neurons is here set to 1 (*I* = *P* = 1), representing that the single feature (ReHo) is used and only one value is obtained for an iteration. The number of hidden layer unit depends as follows:

(3) $$\begin{array}{c} H=\sqrt{I+P}+\alpha , \end{array}$$

where *α* is a constant from 1 to 10. In this study, according to the optimum results of the model iteration, *H* equals 4.

#### 2.2.3. Determining Model Expressions through BPNN Training

The BPNN algorithm need to repeat two stages: forward propagation and reverse propagation.

*Stage 1: forward propagation*. During the forward propagation, ReHo of region A is used to predict ReHo of region B. The detailed process is as follows.

First is setting the training parameters (maximum of training times *ε*=50000, minimum of target error *γ* = 10 − 3, and the learning rate *η*=0.05) and the initial weight of each layer, with *w*_*ih*_ and *w*_*hp*_ to a random value ranged from -1 to 1, which will be adjusted iteratively during training.

Second is establishing the nonlinear mapping from the input layer to the hide layer and the nonlinear mapping from the hide layer to the output layer by formulas (4)–(8).

(4) $$\begin{array}{c} Y_{h}^{A⟶B}=\overset{I}{\underset{i=1}{\sum }}w_{ih}^{A⟶B}X_{i}^{A}\left(h=1,2,\cdots ,H\right), \end{array}$$

(5) $$\begin{array}{c} Z_{h}^{A⟶B}=f\left(Y_{h}^{A⟶B}\right)=\frac{1}{1+\exp\left(-Y_{h}^{A⟶B}\right)}=\frac{1}{1+\exp\left(-\sum_{i=1}^{I}w_{ih}^{A⟶B}X_{i}^{A}\right)}\left(h=1,2,\cdots ,H\right), \end{array}$$

where *I* and *H* are the number of neurons in the input layer and the hide layer, respectively; *Y*_*h*_^*A*⟶*B*^ and *Z*_*h*_^*A*⟶*B*^ are the input value and output value of the *h*-th neuron in the hide layer, respectively; *X*_*i*_^*A*^ is the input value of the *i*-th neuron in the input layer; and *w*_*ih*_^*A*⟶*B*^ is the weight value between the *i*-th neuron of the input layer and the *h*-th neuron of the hide layer. The excitation function *f*(*X*) is a sigmoid function, as follows:

(6) $$\begin{array}{c} f\left(X\right)=\frac{1}{1+e^{-X}}, \end{array}$$

(7) $$\begin{array}{c} Q_{h}^{A⟶B}=\overset{H}{\underset{h=1}{\sum }}w_{hp}^{A⟶B}Z_{h}^{A⟶B}\left(p=1,2,\cdots ,P\right), \end{array}$$

(8) $$\begin{array}{c} \psi_{p}^{A⟶B}=f\left(Q_{p}^{A⟶B}\right)=\frac{1}{1+\exp\left(-Q_{p}^{A⟶B}\right)}=\frac{1}{1+\exp\left(-\sum_{h=1}^{H}w_{hp}^{A⟶B}Q_{p}^{A⟶B}\right)}\left(p=1,2,\cdots ,P\right), \end{array}$$

where *H* and *P* are the number of neurons in the hide layer and the output layer, respectively; *Q*_*p*_^*A*⟶*B*^ and *ψ*_*p*_^*A*⟶*B*^are the input value and output value of the *p*-th neuron in the output layer, respectively; *w*_*hp*_^*A*⟶*B*^is the weight value between the *h*-th neuron of the hide layer and the *p*-th neuron of the output layer; and *f*(*X*)is the same as above.

Note that the parameters of the above process are the intermediate values during the training process of the brain region A predicting the brain region B.

*Stage 2: reverse propagation*. During the reverse propagation, the weight values among the input layer, the hide layer, and the output layer are updated continuously based on the predictive values during stage1, as follows.

First is expressing the real results of the brain region B as REA^*B*^ = (REA_1_^*B*^, REA_2_^*B*^, ⋯, REA_*p*_^*B*^), and then, the predictive error function can be shown as follows:

(9) $$\begin{array}{c} {\text{ERR}}^{A⟶B}=\frac{1}{2}\overset{P}{\underset{p=1}{\sum }}{\left({\text{ERR}}_{p}^{A⟶B}\right)}^{2}{\text{ERR}}^{A⟶B}=\frac{1}{2}\overset{P}{\underset{p=1}{\sum }}{\left(\Psi_{p}^{A⟶B}-{\text{REA}}_{p}^{B}\right)}^{2}=\frac{1}{2}\overset{P}{\underset{p=1}{\sum }}{\left(\frac{1}{1+\exp\left(-\sum_{h=1}^{H}w_{hp}^{A⟶B}Q_{p}^{A⟶B}\right)}-{\text{REA}}_{p}^{B}\right)}^{2}, \end{array}$$

where ERR^*A*⟶*B*^ is the error function of the brain region A predicting the region B; *H* and *P* are the number of neurons in the hide layer and the output layer, respectively; *Q*_*p*_^*A*⟶*B*^ and *ψ*_*p*_^*A*⟶*B*^ are the input value and output value of the *p*-th neuron in the output layer, respectively; and *w*_*hp*_^*A*⟶*B*^ is the weight value between the *h*-th neuron of the hide layer and the *p*-th neuron of the output layer.

Second is calculating the partial derivative of the error function with respect to the weight between the hidden layer and the output layer, as shown as follows:

(10) $$\begin{array}{c} \frac{\partial {\text{ERR}}_{hp}^{A⟶B}}{\partial w_{hp}^{A⟶B}}=\frac{\partial {\text{ERR}}_{hp}^{A⟶B}}{\partial \psi_{p}^{A⟶B}}.\frac{\partial \psi_{p}^{A⟶B}}{\partial w_{hp}^{A⟶B}}=\frac{\partial {\text{ERR}}_{hp}^{A⟶B}}{\partial \psi_{p}^{A⟶B}}\frac{d\psi_{p}^{A⟶B}}{{dQ}_{p}^{A⟶B}}\frac{\partial Q_{p}^{A⟶B}}{\partial w_{hp}^{A⟶B}}=\frac{\partial \left(\left(1/2\right)\sum_{p=1}^{P}{\left(\psi_{p}^{A⟶B}-{\text{REA}}_{p}^{B}\right)}^{2}\right)}{\partial \psi_{p}^{A⟶B}}\frac{d\left[f\left(Q_{p}^{A⟶B}\right)\right]}{{dQ}_{p}^{A⟶B}}\frac{\partial \left(\sum_{h=1}^{H}w_{hp}^{A⟶B}Z_{h}^{A⟶B}\right)}{\partial w_{hp}^{A⟶B}}=\psi \left({}_{p}^{A⟶B}-{\text{REA}}_{p}^{B}\right)f^{\prime }\left(Q_{p}^{A⟶B}\right)Z_{h}^{A⟶B}, \end{array}$$

where ERR^*A*⟶*B*^ is error function; *H* and *P* are the number of neurons in the hide layer and the output layer, respectively; *Q*_*p*_^*A*⟶*B*^ and*ψ*_*p*_^*A*⟶*B*^ are the input value and output value of the *p*-th neuron in the output layer, respectively; *Z*_*h*_^*A*⟶*B*^ is output value of the *h*-th neuron in the hide layer; and *w*_*hp*_^*A*⟶*B*^ is the weight value between the *h*-th neuron of the hide layer and the *p*-th neuron of the output layer.

Third is calculating the partial derivative of the error function with respect to the weight between the input layer and the hidden layer, as shown as follows:

(11) $$\begin{array}{c} \frac{\partial {\text{ERR}}^{A⟶B}}{\partial w_{ih}^{A⟶B}}=\frac{\partial \text{ERR}}{\partial \psi_{p}^{A⟶B}}\frac{d\psi_{p}^{A⟶B}}{{dQ}_{p}^{A⟶B}}\frac{\partial Q_{p}^{A⟶B}}{\partial Z_{h}^{A⟶B}}\frac{{dZ}_{h}^{A⟶B}}{{dY}_{h}^{A⟶B}}\frac{\partial Y_{h}^{A⟶B}}{\partial w_{ih}^{A⟶B}}=\frac{\partial \left(\left(1/2\right)\sum_{p=1}^{P}{\left(\psi_{p}^{A⟶B}-{\text{REA}}_{p}^{B}\right)}^{2}\right)}{\partial \psi_{p}^{A⟶B}}\frac{d\left[f\left(Q_{p}^{A⟶B}\right)\right]}{{dQ}_{p}^{A⟶B}}\frac{\partial \left(\sum_{h=1}^{H}w_{hp}^{A⟶B}Z_{h}^{A⟶B}\right)}{\partial Z_{h}^{A⟶B}}\frac{d\left[f\left(Y_{h}^{A⟶B}\right)\right]}{{dY}_{h}^{A⟶B}}\frac{\partial \left(\sum_{i=1}^{I}w_{ih}^{A⟶B}X_{i}^{A}\right)}{\partial w_{ih}^{A⟶B}}=\left(\psi_{p}^{A⟶B}-{\text{REA}}_{p}^{B}\right)f^{\prime }\left(Q_{p}^{A⟶B}\right)w_{hp}^{A⟶B}f^{\prime }\left(Y_{h}^{A⟶B}\right)X_{i}^{A}, \end{array}$$

where ERR^*A*⟶*B*^ is error function; *I*, *H*, and *P* are the number of neurons in the input layer, hide layer, and the output layer, respectively; *Q*_*p*_^*A*⟶*B*^ and *ψ*_*p*_^*A*⟶*B*^ are the input value and output value of the *p*-th neuron in the output layer, respectively; *Y*_*h*_^*A*⟶*B*^and *Z*_*h*_^*A*⟶*B*^ are the input value and output value of the *h*-th neuron in the hide layer, respectively; *X*_*i*_^*A*^ is the input value of the *i*-th neuron in the input layer; *w*_*hp*_^*A*⟶*B*^ is the weight value between the *h*-th neuron of the hide layer and the *p*-th neuron of the output layer; and *w*_*ih*_^*A*⟶*B*^ is the weight value between the *i*-th neuron of the input layer and the *h*-th neuron of the hide layer.

Fourth is adjusting the weight during iteration according to the error function as follows:

(12) $$\begin{array}{c} w_{hp}^{A⟶B}\left(\theta +1\right)=w_{hp}^{A⟶B}\left(\theta \right)-\eta \frac{\partial {\text{ERR}}^{A⟶B}}{\partial w_{hp}^{A⟶B}}, \end{array}$$

(13) $$\begin{array}{c} w_{ih}^{A⟶B}\left(\theta +1\right)=w_{ih}^{A⟶B}\left(\theta \right)-\eta \frac{\partial {\text{ERR}}^{A⟶B}}{\partial w_{ih}^{A⟶B}}, \end{array}$$

where *θ* represents the *θ*‐th iteration.

Note that similar to stage 1, the parameters of above process are the intermediate values during the training process from the brain region A predicting the brain region B.

Finally, computing the global error as formula (14) and judging whether or not it meets the conditions of termination (global error ≤ *γ* or times of learning≥ *ε*). Once anyone is met, the optimal model of the BPNN can be determined.

(14) $$\begin{array}{c} \text{GE}=\frac{1}{M}\overset{M}{\underset{m=1}{\sum }}\sqrt{\overset{P}{\underset{p=1}{\sum }}{\left(\psi_{p}^{m}-{\text{REA}}_{p}^{m}\right)}^{2}}, \end{array}$$

where *M* is the number of sample, *P* is the number of output neuron in the output layer, *ψ*_*p*_^*m*^ is the output value of *p*-th neuron in the output layer of the *m*-th sample, and REA_*p*_^*m*^ is the real value of *p*-th neuron in the output layer of the *m*-th sample.

Notably, the above process is carried out using the leave-one-subject-out cross validation (LOOCV). In other words, only one subject is left as the test sample for each iteration, and the other *M*-1 samples form the training set. Therefore, *M* iterations are performed in the current study.

#### 2.2.4. EC Measurement

After determining the optimal model of BPNN in the last section, the calculation of EC is performed as the following steps.

First is predicting the results (ReHo) of all brain regions by each another for test sample during each iteration according to the final model as follows:

(15) $$\begin{array}{c} \psi^{A⟶B}=\frac{1}{1+\exp\left(-\right.\sum_{h=1}^{H}w_{h1}^{A⟶B}\left(\sum_{h=1}^{H}w_{h1}^{A⟶B}\left(1/\left(1+\exp\left(-\left(w_{1h}^{A⟶B}X_{1}^{A}\right)\right)\right)\right)\right)}, \end{array}$$

where *ψ*^*A*⟶*B*^represents the predicting result from the brain region A to the brain region B, *w*_1*h*_^*A*⟶*B*^represents the optimal weight between the input value and the *h*-th hide neuron in the hide layer during training from the brain region A to the brain region B, *w*_*h*1_^*A*⟶*B*^represents the optimal weight between the *h*-th hide neuron of the hide layer and the output neuron, and *X*_1_^*A*^represents the ReHo value of brain region A.

Second is calculating the predicted error matrix between any two brain regions, as shown as follows:

(16) $$\begin{array}{c} E^{A⟶B}=\left|\Psi^{A⟶B}-{\text{ReHo}}^{B}\right|, \end{array}$$

(17) $$\begin{array}{c} E=\left[\begin{array}{ccccc} E^{1⟶1} & \cdots & E^{1⟶B} & \cdots & E^{1⟶N}\\ \vdots & \cdots & \cdots & \cdots & \vdots \\ E^{A⟶1} & \cdots & E^{A⟶B} & \cdots & E^{A⟶N}\\ \vdots & \cdots & \cdots & \cdots & \vdots \\ E^{N⟶1} & \cdots & E^{N⟶B} & \cdots & E^{N⟶N} \end{array}\right], \end{array}$$

where *E*^*A*⟶*B*^indicates the error value of brain region A predicting brain region B, *ψ*^*A*⟶*B*^ indicates the finally predicting result from the brain region A to the brain region B, ReHo^*B*^ indicates the real ReHo value of brain region B, and *E* indicates the prediction error matrix.

Third is normalizing and converting the prediction error matrix *E* into the prediction accuracy rate matrix ACC as follows:

(18) $$\begin{array}{c} \begin{array}{c} \text{ACC}=1-\frac{E-\text{MIN}\left(E\right)}{\mathrm{MAX}\left(E\right)-\text{MIN}\left(E\right)},\\ =\left[\begin{array}{ccccc} \text{ACC}1⟶1 & \cdots & \text{ACC}1⟶B & \cdots & \text{ACC}1⟶N\\ \vdots & \cdots & \vdots & \cdots & \vdots \\ \text{ACC}A⟶1 & \cdots & \text{ACC}A⟶B & \cdots & \text{ACC}A⟶N\\ \vdots & \cdots & \vdots & \cdots & \vdots \\ \text{ACC}N⟶1 & \cdots & \text{ACC}N⟶B & \cdots & \text{ACC}N⟶N \end{array}\right], \end{array} \end{array}$$

where ACC_*A*⟶*B*_ indicates the accuracy rate of the brain region A predicting the ReHo of the brain region B, and it ranges from 0 to 1; *E*indicates the prediction error matrix; MAX(*E*) is the maximum value of the error matrix *E*; and MIN(*E*) is the minimum value of the error matrix *E*.

Finally, judging the causal relationship according to the matrix with prediction accuracy rate. For the element of the matrix with prediction accuracy rate, the larger the value, the stronger the causal relationship. Therefore, we define ACC matrix of ReHo as the EC matrix of BPNN, and then, ACC_*A*⟶*B*_ is the effective connectivity EC_*A*⟶*B*_, representing the influence of the brain region A on the brain region B. In the study, if EC_*A*⟶*B*_ is greater than or equal to 0.6 and also less than 0.7, there is a possible causal influence from A to B. In the same way, the 0.7 ≤ result < 0.8, 0.8 ≤ result < 0.9, and 0.9 ≤ result ≤ 1 represent the moderate, good, and excellent causal influence from A to B.

### 2.3. Data and Preprocessing

Simulation data and fMRI data were used here for clarifying the ability of the proposed EC-BP method to evaluate causal relationship.

#### 2.3.1. Simulation Data

Four time series with a length of 200 are simulated, as shown as follows:

(19) $$\begin{array}{c} \left\{\begin{array}{c} X1\left(i\right)=i*\left(4-4*i\right),\\ X2\left(i\right)=X2\left(i\right)*\left(0.5-0.5.*X1\left(i\right)\right)+X1\left(i\right),\\ X3\left(i\right)=X3\left(i\right)*\left(2.1+0.4.*X1\left(i\right)\right)+X1\left(i\right),\\ X4\left(i\right)=X4\left(i\right)*\left(0.8-0.35.*X1\left(i\right)\right)+X1\left(i\right), \end{array}\right. \end{array}$$

where *i* is from 1 to 200.

Formula (19) tells us that the actual causal relationship of these time series is *X*1⟶*X*2, *X*1⟶*X*3, *X*1⟶*X*4. To evaluate the performance of the proposed EC-BP, the mean value of the time points was used as the feature of each time series.

#### 2.3.2. fMRI Data

This study included 60 healthy subjects, who all provided the written informed consent. All participants were interviewed and evaluated by a clinician according to the clinical health standards. Their brain fMRI data during resting state was collected based on 3T MRI scanner (GE Healthcare, Milwaukee, WI, USA). The parameters of the scanning using Echo Planar Imaging are as follows: layer thickness = 5 mm, matrix size = 64 × 64, echo time = 30 ms, repetition time = 2 s, flip angle = 90°, intralayer resolution = 3.75 × 3.75 mm^2^, number of slices = 32, number of volumes = 180, and scan time = 6 minutes. During the scan, the subjects were asked to keep their eyes closed, awake, and relaxed.

The processing of fMRI data before EC-BP are shown by the following two steps: first is carrying out the preprocessing using DPABI (http://rfmri.org/DPABI) [32] developed by the Institute of Psychology of the Chinese Academy of Sciences. The major steps include converting data format from DICOM format to NIFTI format, removing the first 10 time points of every time series, taking slice timing, correcting head motion, normalizing to standard space (MNI space), eliminating linear drift, and performing band-pass filtering (0.01-0.08 Hz). Second is dividing the whole brain into 90 brain regions based on the AAL template after removing the cerebellar structures [33].

## 3. Results

### 3.1. Simulation Data

GCA and the proposed EC-BP were used to evaluate EC of simulation data, and the results are shown in Table 1. Table 1 shows that GCA approach found small EC values for any pair from four time sequences, and the biggest value was the causal value from X2 to X1 (0.0241). The result of GCA is deviated from that of the actual data, being unsuitable for nonlinear causality. However, EC-BP showed more than 0.7 prediction accuracy from X1 to X2 and X3 to X4, and the rest were less than 0.6.

### 3.2. Imaging Data

In this study, the demographic information of all healthy subjects is shown in Table 2.

Table 3 shows the strongest 50 ECs using EC-BP and GCA, which exhibited a clear difference between both methods. The strong ECs found by EC-BP were directionally from multiple regions to parahippocampal (PHG) and hippocampus (HIP), whereas the strong EC found by GCA was mainly connected to the paracentral lobule (PCL, 22/50) and basal ganglia network (BG) (18/50) including caudate nucleus (CAU), pallidum (PAL), and putamen (PUT) and partly connected to the middle temporal gyrus (MTG), supplementary motor area (SMA), precentral gyrus (preCG), precuneus (PCUN), and thalamus (THAL).

**Figure 2:**
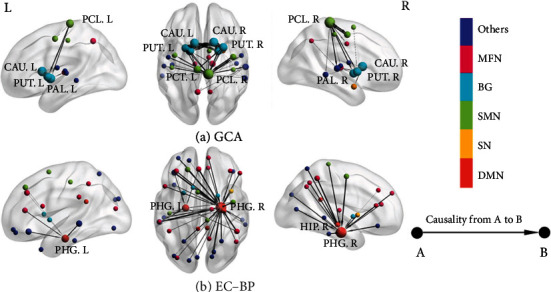
Strongest 50 effective connections (ECs) obtained by different methods.

Figure 2 also shows the distribution of brain areas related to the strongest 50 ECs found by both methods. EC-BP is involved in broader regions (frontal and occipital cortices) compared with GCA. Moreover, ECs detected by EC-BP were all related to default mode network (DMN), sensorimotor network (SMN), multifunctional network (MFN), and other networks, whereas ECs detected by GCA were mainly related to the basal ganglia (BG), SMN, MFN, and other networks. The brain is divided into different networks according to the reports by Shirer et al. [34].

## 4. Discussion

In this study, we proposed a new method for measuring brain EC, named “EC-BP,” which is performed based on BPNN prediction and LOOCV. It overcomes the disadvantages of the existing methods measuring brain EC, which do not require any prior knowledge. Moreover, simulation data confirms that it is better than the current common method (GCA) to detect the nonlinear causal relationship. Furthermore, our investigations about brain fMRI of healthy subjects show that the proposed method is a “good” addition to the existing method on evaluating the brain EC.

The traditional methods measuring brain EC (SEM, MAR, DCM, and GCA) mainly use the initial time series, which are relatively intuitive but sensitive to the fluctuation of each time point. Therefore, they are affected easily by noise. Relative to them, the proposed EC-BP is carried out based on the higher-level features, which is more robust to noise.

The results from the four simulated time series showed that the proposed EC-BP can detect the nonlinear causal relationship, consistent with the actual causality. However, the GCA can not do that. This is mainly due to the procedure of the GCA method, which assumed that the detected signals had linear characteristics. As we all known, the brain functional signal is nonlinear, and the correlation between different brain regions is not limited to linear relationship, which may be affected by third-party region. In contrast, the EC-BP method is based on a neural network prediction model and detects the effective connectivity through a nonlinear mapping relationship, which will provide more advantages for detecting the functionally nonlinear relationship between the brain region activities. Therefore, the proposed method may avoid the omissive causality caused by the GCA, which has great value for the analysis of fMRI data.

Based on the analysis of the fMRI data from the healthy participants, EC-BP and GCA showed the huge differences in the top 50 connections in descending order of EC. EC-BP showed the directed connections linked to HIP/PHG where hemodynamic activity varies nonlinearly with multiple regions, whereas GCA showed many connections related to the PCL, CAU, PUT, and PAL where hemodynamic activity varies linearly with multiple regions. The above brain regions exhibit most frequent information transfer measured by different methods. As we know, the HIP and PHG have the important contribution of human memory. As Opitz summarized [35], the HIP plays a vital role in the memory associated with the recollection-based cognition, retrieval of contextual information, and unique conjunction of face-voice and object-location associations. Moreover, the hippocampus can also promote the combination of different cortical representations. That is to say, two inputs, coming from disparate neurons but being composed into a bound representation, activate the same neurons within the HIP, thereby resulting in distinct representations. The PHG, located inferior to the HIP, was interacted with the HIP, enhancing memory performance. Therefore, higher EC of the HIP/PHP in our results may be suggestive of their flexibility that they can be capable to rearrange any arbitrary relation, bind the separated cortices, and reconstruct single input from multiple neurons. The PCL straddles at the boundary between the frontal lobe and parietal lobe, referring to the primary motor and sensory areas relevant to the parts of limbs. Our findings about the higher EC of the PCL may be concerned with the scanning process and environment. The PAL, CAU, and PUT are the important parts of the BG in higher vertebrates [36]. As Simonyan summarized [37], the intrinsic connectivity of the BG can include both direct and indirect pathways, involved in cortico-BG-thalamo-cortical loop, while the extrinsic connectivity of the BG can refer to three functional loops including the motor loop via motor/premotor cortices, the associative loop via the dorsolateral part of the prefrontal cortex and parietal cortex, and the limbic loop via the orbital and medial parts of the prefrontal cortex. The interaction and integration of the above three loops may contribute to the complex functions of the BG, including working memory, emotions, decision-making, language, and procedural learning. Therefore, the higher EC found by GCA may suggest the functional importance of the BG within the brain functional network during resting state. Above all, a lot of communication within the healthy brain may be directly linked to the BG, whereas the activities of many brain regions may need to nonlinearly depend on the memory from HIP/PHG for healthy people during the resting state.

Given the results of Figure 2, EC-BP found lots of areas linked to DMN for the strongest 50 ECs. DMN was discovered by Shulman et al. in 1997 [38] and was first proposed by Raichle et al. in 2001 with PET technology [39]. It refers to the areas with significant higher spontaneous activity during resting state. The current results obtained by EC-BP found that a large number of brain regions have certain effects on DMN, which may be the reason why those regions within DMN are higher active than other brain regions during the resting state. However, BG, as the important parts of the strongest 50 ECs found by GCA, was not in the top 50 for EC-BP. This indicated that the nonlinear causality related to DMN may be stronger than the linear causality relevant to BG for the healthy subjects during the resting state. Given the above analysis, the proposed EC-BP method can provide supplementary information to GCA, which will promote more comprehensive detection and evaluation of brain EC.

However, there are some limitations in our study. (i) In the study, single feature (ReHo was selected for our preliminary attempt) was extracted for performing EC-BP. Actually, the more features, the more accurate the results are likely to be achieved. Therefore, in the future study, more features or indicators should be included. (ii) The proposed method needs to be validated in a variety of brain-related clinical disease. The disease should be associated with aberrations of the EC, and the results of the classification between the patients and controls can be compared to further prove the effectiveness of the method.

## 5. Conclusions

The proposed EC-BP method can provide supplementary information to GCA, which will promote more comprehensive detection and evaluation of brain EC.

## Figures and Tables

**Table 1:** Values of effective connections (ECs) obtained by EC-BP and GCA.

Our method	X1	X2	X3	X4	GCA	X1	X2	X3	X4
X1	1	0.721	0.723	0.727	X1	NaN	0.016	0.012	0.012
X2	0.528	1	0.524	0.527	X2	0.0241	NaN	0.028	0.022
X3	0.332	0.327	1	0.332	X3	0.003	0.010	NaN	0.001
X4	0.136	0.129	0.130	1	X4	0.001	0.000	0.000	NaN

The EC value in this table represents the causal relationship from the corresponding row to the corresponding column. X1, X2, X3, and X4 are four time sequences. EC-BP: effective connectivity based on back-propagation neural network method; GCA: Granger causality analysis.

**Table 2:** Demographic information of healthy participants.

Items	Value
Age (years)	22.35 ± 1.0928
Gender (male/female)	23/37
Height (cm)	162.2833 ± 7.4254
Weight (kg)	52.4833 ± 7.4465

**Table 3:** The top 50 effective connections in descending order for EC-BP and GCA.

EC-BP			GCA		
Effective connections		EC_*A*⟶*B*_ value	Effective connections		EC_*A*⟶*B*_ value
Region A	Region B		Region A	Region B	
MTG.L	PHG.R	0.7642	CAU.R	MTG.R	0.1479
ORBinf.L	PHG.R	0.7575	PreCG.L	PCL.L	0.1485
ACG.L	PHG.R	0.7551	PreCG.R	PCUN.L	0.1495
IPL.R	PHG.R	0.7533	MTG.L	PCL.R	0.1516
PreCG.R	PHG.R	0.7512	PUT.R	SMA.R	0.1516
SFGdor.L	PHG.R	0.7437	HES.R	PCL.R	0.1522
MFG.L	PHG.R	0.7437	AMYG.R	CAU.L	0.1535
ORBmid.L	PHG.R	0.7421	STG.R	PCUN.R	0.1545
SPG.R	PHG.R	0.7417	MTG.R	PCL.R	0.1569
ITG.R	PHG.R	0.7408	HES.L	PCL.R	0.1574
PCL.R	PHG.R	0.7406	STG.R	PCUN.L	0.1577
PCUN.R	PHG.R	0.7392	PUT.R	THA.R	0.1594
IFGtriang.R	PHG.R	0.7386	PAL.R	THA.R	0.1595
ORBinf.R	PHG.R	0.7384	CAU.L	PUT.R	0.1616
CAL.R	PHG.R	0.7382	ROL.R	PCL.R	0.163
ITG.L	PHG.R	0.7381	PCL.R	PreCG.R	0.1632
SFGmed.R	PHG.R	0.7377	PAL.L	THA.L	0.1647
CUN.R	PHG.R	0.7369	PoCG.R	PCL.R	0.1656
INS.R	PHG.R	0.7364	CAU.L	PAL.R	0.166
CAU.L	PHG.R	0.7362	PAL.L	THA.R	0.1666
MTG.L	PHG.L	0.7356	DCG.R	PCL.R	0.168
SPG.L	PHG.R	0.7348	AMYG.R	CAU.R	0.1691
SMA.L	PHG.R	0.7313	STG.L	PCL.R	0.1704
IFGoperc.L	PHG.R	0.7312	PAL.L	PCL.L	0.1785
ACG.R	PHG.R	0.73	PUT.R	PCL.L	0.1795
TPOmid.L	PHG.R	0.73	PoCG.L	PCL.R	0.1796
ORBinf.L	PHG.L	0.729	PAL.R	PCL.L	0.1797
SMG.L	PHG.R	0.728	CAU.L	PUT.L	0.1846
ANG.R	PHG.R	0.7273	ROL.L	PCL.R	0.187
ACG.L	PHG.L	0.7265	CAU.R	PUT.R	0.187
MTG.R	PHG.R	0.7254	PUT.L	PCL.R	0.1871
SOG.L	PHG.R	0.725	PAL.L	PCL.R	0.1901
PoCG.L	PHG.R	0.7249	PUT.R	PCL.R	0.1901
IPL.R	PHG.L	0.7248	PreCG.L	PCL.R	0.1903
PAL.R	PHG.R	0.7241	CAU.R	PUT.L	0.1905
PreCG.R	PHG.L	0.7226	CAU.R	PAL.R	0.1949
PCG.L	PHG.R	0.7224	STG.R	PCL.R	0.1954
MTG.L	HIP.R	0.7222	PUT.L	PCL.L	0.1958
MOG.L	PHG.R	0.7219	PUT.R	CAU.L	0.1967
CUN.L	PHG.R	0.7215	CAU.L	PAL.L	0.1972
IPL.L	PHG.R	0.7211	PAL.R	PCL.R	0.2006
THA.R	PHG.R	0.7208	CAU.R	PAL.L	0.2065
PCG.R	PHG.R	0.7188	PAL.R	CAU.L	0.2075
ORBsup.L	PHG.R	0.7172	PUT.L	CAU.R	0.217
PUT.L	PHG.R	0.717	PUT.L	CAU.L	0.2203
ORBinf.L	HIP.R	0.7156	PUT.R	CAU.R	0.2217
IOG.L	PHG.R	0.7154	PreCG.R	PCL.R	0.2306
SFGdor.L	PHG.L	0.7152	PAL.R	CAU.R	0.2377
MFG.L	PHG.L	0.7152	PAL.L	CAU.R	0.2378
MFG.R	PHG.R	0.7143	PAL.L	CAU.L	0.246

All regional abbreviations are from AAL template. EC: effective connectivity; EC-BP: effective connectivity based on back-propagation neural network method; GCA: Granger causality analysis.

## Data Availability

The datasets used and analyzed during the current study are available from the corresponding author on reasonable request.
